# Condition “Vasa Vasorum” in Patients with Thoracic Aortic Aneurysm

**DOI:** 10.3390/jcm12103578

**Published:** 2023-05-20

**Authors:** Petr V. Chumachenko, Alexandra G. Ivanova, Mariam Bagheri Ekta, Andrey V. Omelchenko, Vasily N. Sukhorukov, Alexander M. Markin, Yuliya V. Markina, Anton Y. Postnov

**Affiliations:** 1 Petrovsky National Russian Research Center of Surgery, Lane Abrikosovsky, 2, 119991 Moscow, Russia; chumach7234@mail.ru (P.V.C.); gussona@gmail.com (A.G.I.); ms.bvgheri@gmail.com (M.B.E.); vnsukhorukov@gmail.com (V.N.S.); alexander.markin.34@gmail.com (A.M.M.); yu.v.markina@gmail.com (Y.V.M.); 2 National Medical Research Center of Cardiology, Academician Chazova St., 15a, 121552 Moscow, Russia; 3 Institute of General Pathology and Pathophysiology, Baltiyskaya St., 8, 125315 Moscow, Russia; omi@bk.ru; 4 Peoples’ Friendship University of Russia Named after Patrice Lumumba (RUDN University), Miklukho-Maklaya St., 6, 117198 Moscow, Russia

**Keywords:** aortic aneurysm, vasa vasorum, inflammatory infiltrates, immunohistochemistry

## Abstract

It is known that vasa vasorum contributes substantially to the blood supply and nutrition of one-third of the wall of the ascending thoracic aorta. Therefore, we focused on studying the relationship between inflammatory cells and vasa vasorum vessels in patients with aortic aneurysm. The material for the study was biopsies of thoracic aortic aneurysms taken from patients during an aneurysmectomy (34 men, 14 women, aged 33 to 79 years). The biopsies belonged to patients with non-hereditary thoracic aortic aneurysm. An immunohistochemical study was carried out using antibodies to antigens of T cells (CD3, CD4, CD8); macrophages (CD68); B cells (CD20); endothelium (CD31, CD34, von Willebrand factor (vWF)); and smooth muscle cells (alpha actin). Samples without inflammatory infiltrates contained less vasa vasorum in the tunica adventitia than samples with inflammatory infiltrates, and this difference was statistically significant *p* < 0.05. T cell infiltrates in the adventitia of aortic aneurysms were found in 28 of 48 patients. In the vessels of the vasa vasorum, surrounded by inflammatory infiltrates, T cells that adhered to the endothelium were found. The same cells were also localized in the subendothelial area. The number of adherent T cells in patients with inflammatory infiltrates in the aortic wall dominated the number of these cells in patients without inflammation of the aortic wall. This difference was statistically significant, *p* < 0.0006. Hypertrophy and sclerosis of the arteries of the vasa vasorum system, the narrowing of their lumen, and, as a result, impaired blood supply to the aortic wall, were found in 34 patients with hypertension. In 18 patients (both in patients with hypertension and in patients without hypertension), T cells that adhered to the vasa vasorum endothelium were found. In nine cases, massive infiltrates of T cells and macrophages were found, which surrounded and squeezed the vasa vasorum, preventing blood circulation. In six patients, parietal and obturating blood clots were found in the vasa vasorum vessels, which disrupted the normal blood supply to the aortic wall. We believe that this indicates the importance of the state of the vessels of the vasa vasorum in the development of an aortic aneurysm. In addition, pathological changes in these vessels may not always play a primary role, but always a very important role, in the pathogenesis of this disease.

## 1. Introduction

Vasa vasorum dysfunction and the presence of inflammatory infiltrates in the aortic wall are the risk factors contributing to an aortic aneurysm. These processes are constantly interacting with each other. In a study, Osada H. et al. (2013) examined samples of aortic aneurysm taken from 21 patients and concluded that damage to vasa vasorum leads to the development of medial necrosis and the subsequent rupture of the aortic aneurysm [1]. Similar conclusions were reached by Angouras D.S. (2013) and Tanaka H. (2017) [2,3]. They blocked the animals’ blood flow through the vasa vasorum, and this led to the rupture of the previously formed aortic aneurysm. The obtained data allowed them to conclude that vasa vasorum damage plays a major role in the development of aortic aneurysm dissection and rupture. Damage to the vasa vasorum leads to the impaired diffusion of nutrients and oxygen into the outer third of the aortic wall, and causes increased stiffness of the outer aortic wall. Therefore, the aorta, from a mechanical point of view, turns into a two-phase medium: internal and external. The inner part of the aorta receives sufficient nutrition and has normal elasticity, while the outer part (which receives nutrition from vasa vasorum) is ischemic and has increased stiffness. Shear stress occurs at the boundary of these media, which ultimately leads to aortic dissection [2]. An important role in pathogenesis is played by inflammatory infiltrates. Expressed matrix metalloproteinases (ММР1, ММР2, ММР9) destroy the elastic and collagen fibers, which leads to the mechanical weakening of the aortic wall [4].

In addition, the association between mononuclear inflammatory cells of the aortic wall and chemokines (tumor necrosis factor-α, interferon-γ, interleukin-1,2, etc.) causes vasa vasorum endothelial dysfunction. Endothelial dysfunction is characterized by the expression of P-selectin and E-selectin, which contribute to the displacement of inflammatory blood mononuclear cells from the middle of the blood flow towards the vascular wall, closer to the endothelium [5,6,7]. Blood mononuclear cells first tether to the luminal side of the blood vessel wall, and then start rolling and transition into a process called “slow rolling”; after that, cells firmly adhere to the wall, and finally T cells transendothelially migrate into the intima [5,6]. It is believed that inflammatory mononuclear cells migrate into the aortic wall from the vasa vasorum from the blood flow of the aorta. It could mean that aortic wall inflammation is characterized by endothelial adhesion and trans endothelial migration from the vasa vasorum to the surrounding tissues. Thus, vasa vasorum play an important role in the pathogenesis of aortic aneurysm. The role of inflammatory mononuclear cells in the aortic wall has been studied, but there are no studies that investigate the relationship of T cells adhered to the vasa vasorum endothelium with inflammatory infiltrates in aortic aneurysm.

The aim of our study was to study the relationship between the state of vasa vasorum and clinical and pathological data, and to compare the number of T cells adhered to the vasa vasorum endothelium with the size of inflammatory infiltrates in the aneurysm wall.

## 2. Materials and Methods

### 2.1. Subjects

For this study, we collected segments of aortic aneurysm wall, consisting of intima, media, and adventitia. The material was obtained during the operation of aortic aneurysm prosthetics. We studied the biopsy material of the thoracic aorta of patients with a clinical diagnosis of thoracic aortic aneurysm. Specimens were collected from 34 men and 14 women. The age of patients ranged from 33 to 79 years. Only the medial layer and adventitia of the aortic aneurysm were examined. In total, of the 48 patients with aortic aneurysm, 34 patients had hypertension, 7 patients had coronary heart disease, 3 patients had chronic obstructive pulmonary disease, and 1 patient had chronic heart failure. The rest of the patients had no comorbidities.

### 2.2. Formaldehyde-Fixed, Paraffin-Embedded Aortic Tissue Sections

Dissected aortic tissue was fixed with 4% formaldehyde for no less than 24 h at room temperature. Further, dissected aortic tissue fixed in formaldehyde was washed with running tap water for 1–5 min. Biopsies about 1.5 × 0.5 × 0.5 cm in size were prepared. Aneurysm biopsies were immediately placed again in 4% formaldehyde for 24 h at room temperature. Then, tissues were rinsed with running tap water for 30 min to eliminate the formaldehyde. Tissues were also dehydrated through 70%, 80%, 95%, and 100% alcohol each 3 times for 5 min. Further, we cleared tissues in xylene 2 times, for 5 min each and immersed tissues in paraffin 3 times, for 5 min each. Prepared sections were suitable for immunochemistry.

### 2.3. Immunostain Formaldehyde-Fixed, Paraffin-Embedded Aortic Tissue Section

Sections were deparaffinized in xylene, passed through alcohols, and endogenous peroxidase was blocked in them with 0.5% H_2_O_2_ (60 min). Deparaffinized sections were placed in an automated immunohistochemistry stainer, the Ventana system (Roche, San Francisco, CA, USA). The Ventana system fully automates the antibody staining. All biopsies (48 paraffin blocks) obtained from 48 patients were stained with antibodies to CD3, CD4 and CD8 T-cells, CD68 macrophages (Roche, USA); anti-Von Willebrand factor antibodies (Sigma Immuno Chemicals, Burlington, MA, USA); endothelial nitric oxide synthase (Santa Cruz Bio-Technology, Dallas, TX, USA); α-smooth muscle cell actin; CD20 and CD34 antibodies (Roche, USA). Antibodies were applied to the section at a concentration of 10 to 50 μg/mL (depending on the antibody clone) in phosphate buffer pH 7.6. The exposure of antibodies on the section did not exceed 30–45 min at room temperature. Antibodies were visualized using an immunoperoxidase reaction in a Ventana immunostainer and in the classical way, according to the generally accepted method.

In the classical setting of the immunoperoxidase reaction, an avidin–biotin complex with peroxidase from the company was used along with a mouse and rabbit specific HRP/DAB (ABC) detection IHC kit. The immunoperoxidase reaction in the Ventana immunostainer was carried out using solutions certified by the company and according to the technology certified by the company. According to the degree of inflammatory activity in the aortic aneurysm wall, all samples were divided into 3 groups (Group 1 with inflammatory activity in the aortic wall, Group 2 with single and small mononuclear cell infiltrates in the adventitia, and Group 3 without inflammatory infiltrates). The average value of the aortic aneurysm diameter in patients of Group 1 was 61.5 mm (from 52 mm to 77 mm), in patients of Group 2 was –58 mm (from 47 mm to 70 mm), and in patients of group was 3–58 mm (from 46 mm to 70 mm). Previously, the thicknesses of the intima, media, and adventitia (in µm) were measured in biopsy sections. Measurements were taken at six different, randomly selected locations (6 intima measurements, 6 medial measurements, and 6 adventitia measurements in each preparation). The statistical processing of data on the thickness of various layers of the aneurysm was performed using the Microsoft Excel 2013 program. The number of T cells on the vasa vasorum endothelium was calculated over the entire area of the aortic biopsy section. We did not count mononuclear cells in the intima and in atherosclerotic plaques in patients with aortic aneurysm. The comparison was made on the basis of the Fisher’s exact test for count data and the Fisher pair test. Differences were considered significant at a confidence level of *p* ≤ 0.05. The study was conducted in accordance with the Declaration of Helsinki, and approved by the Institutional Review Board of Petrovsky National Russian Research Center of Surgery.

## 3. Results

**Figure 1:**
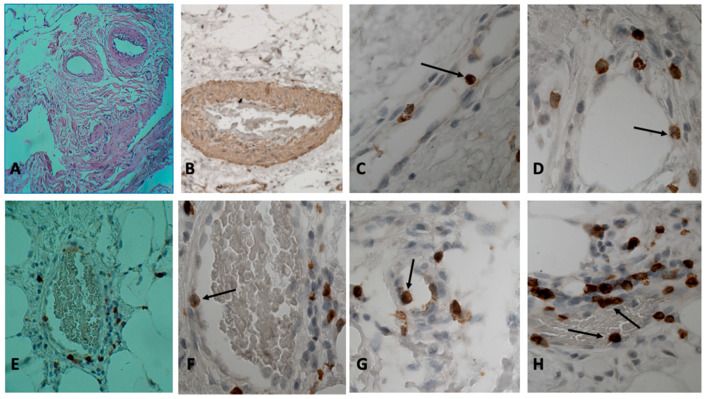
Adventitia of thoracic aortic aneurysm. (**A**) Vasa vasorum in the adventitia of the thoracic aorta. Hypertrophy of the walls of blood vessels. Stained with hematoxylin and eosin. ×200. (**B**) Hypertrophy of the walls of the vessel’s vasa vasorum. Immunohistochemical reaction (IHC) to alpha-actin of smooth muscle cells. Cell nuclei were stained with hematoxylin. ×400. (**C**) Adhered T cells on the endothelium of the vessel vasa vasorum. IHC reaction to CD3 T cells. Cell nuclei were stained with hematoxylin. ×400. (**D**) CD3 T cells adhered to the endothelium of the vessel vasa vasorum. IHC reaction to CD3 T cells. Cell nuclei were stained with hematoxylin. ×600. (**E**) Thin-walled vessel of the vasa vasorum system. In the lumen of the vessel stasis of erythrocytes. Migration of CD4 T cells into the vessel walls of the vasa vasorum. IHC reaction to CD4 T cells. Cell nuclei were stained with hematoxylin. ×400. (**F**) The same preparation, but an endothelial-adhered CD4 T cell. IHC reaction with antibodies to CD4 T cells. Cell nuclei were stained with hematoxylin. ×600. (**G**) CD8 T cell adhered to the endothelium of the vessel vasa vasorum. IHC reaction with antibodies to CD8 T cells. Cell nuclei were stained with hematoxylin. ×600. (**H**) Endothelial-adhered CD8 T cells. Fresh thrombus in the lumen of the vessel. IHC reaction with antibodies to CD8 T cells. Cell nuclei were stained with hematoxylin. ×600.

The histological diagnosis of aortic aneurysm biopsies showed that in the medial layer of the aorta, the bundles of smooth muscle cells in some places had different directions and differed in thickness. Along with the hypertrophy of smooth muscle cells, areas with atrophied smooth muscle cells were observed. In many cases, connective tissue dysplasia was observed, according to the type of cystic media necrosis. In twenty-four patients, re-vascularization of the middle membrane was noted. Twenty-two patients had sections of arterial wall dissection along the middle layer, with the development of common sclerotic changes in all layers. Group 1 consisted of patients in whom abundant inflammatory infiltration of the aortic wall was not limited to the adventitia, but also spread to the medial membrane (see Figure 1A). In Group 2, the inflammatory infiltration of the adventitia was moderate and did not spread to other areas. In Group 3, inflammatory mononuclear cells in the wall of the aortic aneurysm were absent or were detected as single cells in the field of view and were localized only in the adventitia. The measurement of the wall thickness (in μm) of the aortic aneurysm revealed differences in wall thickness in different groups; however, these changes were not statistically significant (adventitia thickness on average in groups ranged from 1076.49 ± 330.72 to 1763.62 ± 1293.49). The thicknesses of the medial membranes were from 1317.74 ± 481.07 to 1945.22 ± 367.36, and the thicknesses of the intima were from 55.55 ± 53.31 to 84.31 ± 56.92.

**Figure 2:**
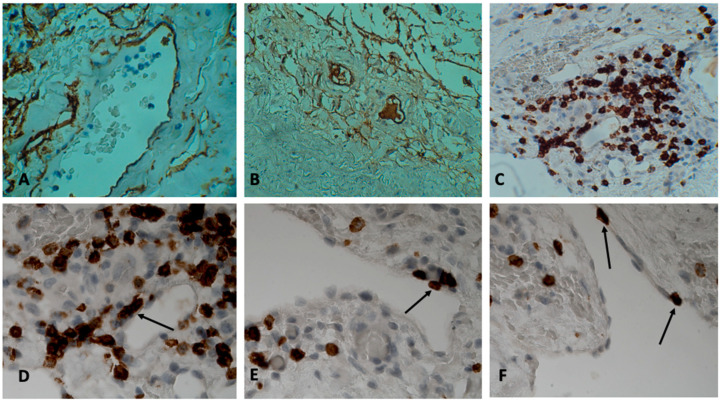
Adventitia of thoracic aortic aneurysm. (**A**) CD34 antigen in the vasa vasorum endothelium. In the lumen of the vessel shadows of erythrocytes and a fresh parietal thrombus. In the surrounding tissue shadows of erythrocytes. Immunohistochemical reaction (IHC) with antibodies to CD34 antigen. Cell nuclei were stained with hematoxylin. ×400. (**B**) Parietal and obturating thrombus in the vessels of the vasa vasorum. IHC reaction with antibodies to von Willebrand factor. Cell nuclei were stained with hematoxylin. ×200. (**C**) CD8 T cell adhered to the endothelium of the vessel vasa vasorum. The surrounding tissue is infiltrated with CD8 T cells. IHC reaction with antibodies to CD8 T cells. Cell nuclei were stained with hematoxylin. ×400. (**D**) CD8 T cell adhered to the endothelium of the vessel vasa vasorum. The surrounding tissue is infiltrated with CD8 T cells. IHC reaction with antibodies to CD8 T cells. Cell nuclei were stained with hematoxylin. ×600. (**E**) T cell adhered to the vessel. IHC reaction with antibodies to CD8 T cells. Cell nuclei were stained with hematoxylin. ×600. (**F**) CD8 T cells adhered to the vascular endothelium of the vasa vasorum system. Cell nuclei were stained with hematoxylin. IHC reaction with antibodies to CD8 T cells. ×600.

In the patients of the first group (number of patients in each group see in Table 1), CD3, CD4, CD8 T cells, and CD68-macrophages were detected immunohistochemically in inflammatory infiltrates. Visually, CD4+ T cells and CD68+ macrophages dominated among the inflammatory mononuclear cells in the infiltrates. It is noteworthy that the frequent localization of T cells and macrophages around the vasa vasorum was observed (9 cases) (Figure 2C). In places where inflammatory infiltrates surrounded the vasa vasorum, T cells that adhered to the endothelium could be seen in the vessel lumen (Figure 1C,D,F–H). Both CD4 and CD8 T cells were in vasa vasorum at different stages of adhesion, for example, 3-stage cell adhesion which involves attachment to the vasa vasorum endothelium (arrest) and the spreading of the adherent T cell on it, and 4-stage adhesion stages of cell migration in the vessel wall (paracellular or transcellular transmigration) [6]. In our opinion, the first two stages of adhesion, the stage of fast-rolling and the stage of slow rolling, are difficult to visualize morphologically [6]. However, the detection of round (in shape) adhesive cells suggests that they are, according to this classification, either at stage 1 or 2 of adhesion.

The adhesive T cells were found only in thin-walled vessels. Most of these vessels contained red blood cells, so they are not lymphatic capillaries, but venules. The blood in these vessels was often in a state of stasis. It should be noted that sometimes T cells used the same areas on the endothelium during adhesion (Figure 2C,D). At the same areas, two or more CD3 T cells could be seen. When introduced into the surrounding tissues, the round shape of the lymphocyte changed to flattened, elongated, or irregular (Figure 1H: adhesive CD8 T cell has a flattened shape, and under it there is another CD8 T cell migrating into the vessel wall). In all patients of the first group (patients with a high activity of the inflammatory process in the aneurysm wall), in the lumen of the vasa vasorum vein and among the attached cells, there were CD3, CD4, and CD8 T cells (Figure 1C–H and Figure 2C–F) (usually one or two T cells were attached in one vein of the vasa vasorum). Almost always, the tissue surrounding this vessel (the vasa vasorum system) contained inflammatory infiltrates composed of the same T cells.

In 50% of patients (7 patients out of 14) (Table 1) with an insignificant degree of activity of the inflammatory process (Group 2), CD3, CD4, and CD8 T cells that adhered to the endothelium were found in the venous, thin-walled vessels of the vasa vasorum system. Usually there were no more than three such cells in the preparation. T cells were at different stages of adhesion. Some of the adherent T cells were located sub endothelially (migration stage) (Figure 1E,F). In one preparation, adhesion phenomena occurred in no more than one or two vasa vasorum. Small infiltrates of CD3, CD4, and CD8 T cells often occurred in the immediate surroundings of such veins. It was not possible to detect adherent T cells in the arteries of the vasa vasorum system (in this group of patients).

T cells that adhered to the endothelium vasa vasorum were found only in 16% of patients (four patients) of Group 3 (patients with no visible inflammatory infiltrates in the wall of the aortic aneurysm). In the vast majority of patients in this group (84% or 21 patients), it was not possible to detect adherent T cells in the veins of the vasa vasorum. It should be noted that these were single T cells in the preparation, and the tissues surrounding these vessels did not contain inflammatory mononuclear cells. In the patients of this group, we failed to detect T cells at the stage of migration into the intima of the vessel. It should be noted that sometimes T cells used the same areas on the endothelium during adhesion (Figure 2D). At the same areas, two or more CD8 T cells could be seen.

Between Group 1 and Group 3, a statistically significant difference was found in the number of T cells that adhered to the vasa vasorum (Table 1). The patients of Group 2 occupied an intermediate position between the groups, which explains our result.

Immunohistochemical analysis with antibodies to the von Willebrand factor and to the CD34 antigen revealed blood thrombosis in the vessels of the vasa vasorum system in some patients (in six patients) (Figure 2A,B). In the vessels of the vasa vasorum system, the phenomena of the stasis of erythrocytes, rupture, and damage to the vessels occurred (Figure 2A). Of the 48 patients, 34 patients had stage 3 hypertension (for 2 or more years). The reaction with antibodies to the alpha-actin of smooth muscle cells revealed hypertrophy of the walls of the arteries of the vasa vasorum system, and hyperplasia of smooth muscle cells in these vessels (Figure 1A,B). In some patients, parietal and even obturating blood clots were found in the vessels of the vasa vasorum (six patients).

## 4. Discussion

The role of vasa vasorum in the pathogenesis of aortic aneurysm has been proven by clinical and animal studies [1,8]. In 2013, it was shown that impaired blood flow in the vasa vasorum system leads to the development of aortic aneurysm and further dissection [1]. This dependence is confirmed by clinical studies, which revealed damage to the vasa vasorum in all patients with aortic aneurysm [1,8,9]. Previous work demonstrated a statistically significant increase in the number of vasa vasorum in patients with aortic dissection [10]. We consider the appearance of numerous newly formed vessels of the vasa vasorum to be the result of a process aimed at compensating for the lack of oxygen in the aortic wall by “creating” the newly formed vasa vasorum. However, the newly formed vasa vasorum is often “not perfect”, and cannot drastically improve the supply of oxygen to the aortic wall. As a result, due to a lack of oxygen, medial necrosis develops in the aortic wall, and, as a result of these processes, dissection and rupture of the aortic aneurysm occur. According to clinical observations, aortic aneurysm dissection often develops in patients with hypertension. It is believed that high aortic pressure leads to vasa vasorum compression, disruption of the blood flow to the aortic wall, and, as a result, to the dissection and rupture of its wall [11,12,13,14]. This is confirmed by the hypertrophy of the walls of the vasa vasorum vessels and the hyperplasia of smooth muscle cells in them (obviously compensatory) found in all patients with stage 3 hypertension. It was previously noted that in patients with aortic aneurysm, there is an increase in smooth muscle cells around the vasa vasorum [15]. It can be assumed that such changes in the walls of the vasa vasorum are inevitably accompanied by a vessel lumen diameter reduction, which leads to an even greater disruption of the blood supply to the aortic wall. In addition, in these patients, parietal thrombi, thrombi obturating the lumen, perivascular inflammatory infiltrates, and adhesive T cells in venous (thin-walled) vasa vasorum were all found. All of the above also negatively affect the blood flow to the aortic wall.

Although 14 out of 48 patients with thoracic aortic aneurysm did not suffer from hypertension, they also had damage to the vasa vasorum: parietal thrombus and obturating thrombus were found; blood vessel ruptures with perivascular hemorrhages; perivascular inflammatory infiltrates; and endothelial adherent T cells. Cell counting (in all 48 patients) of T cells that adhered to the endothelium showed a statistically significant difference in the number of T cells (*p* < 0.0006) in patients with massive inflammatory infiltrates of the aortic wall compared with patients without inflammatory infiltrates in the aorta. In other words, the more massive the inflammatory infiltrates in the aortic wall, the more T cells that adhered to the vasa vasorum. A strict compliance between the number of T cells adhered to the vasa vasorum and the activity of the inflammatory process in the aorta suggests that T cells migrate into the aortic wall (adventitia and approximately 1/3 of the outer part of the media) from the vessels of the vasa vasorum system, and not from the main blood flow of the aorta. The detection of single cells adhered to the vasa vasorum in individual patients (4 out of 25 patients) in the group without inflammation (Group 3) may indicate the immediacy of the detected process, and that this adhesion can be safely resolved and the adhered T cell will again be free in the bloodstream (i.e., perhaps the cell is currently in the first stage of a fast or second stage of a slow rolling). It is morphologically difficult to distinguish the fast-rolling stage from the slow rolling stage.

It should be noted that in patients without inflammation (Group 3), but with single T cells adhered to the vasa vasorum, no T cells were located sub-endothelially, i.e., the cells were not in a state of migration into the surrounding tissues. This proves that the adhesion process at a certain stage is in an equilibrium state, and, with a favorable development of events, the normal state of endothelial cells is restored, which leads to the “disappearance” of adhesion molecules on the surface of the endothelium and the “release” of the adhered cell, without an introduction into the surrounding tissues. On the other hand, adherent inflammatory mononuclear cells found on the endothelium indicate the dysfunction of the endothelium in this area. Dysfunction may be associated with impaired rheology in this vasa vasorum vessel. The violation of the blood circulation to the aortic wall causes media necrosis and contributes to the development of aortic aneurysm dissection [16,17,18].

### The Limitations of the Study

The main limitations of this study were associated with the preliminary selection of patients by surgeons, since only those patients who were indicated for surgical treatment were subjected to the study, and the state of their general health should have allowed them to undergo a serious surgical operation. This explained the relatively small sample size associated with the restrictive criteria adopted. One biopsy sample was examined from one patient, which may limit the accuracy of the results obtained. More data are needed, including studies by other scientists.

In conclusion, we would like to note once again that all 48 patients with aortic aneurysm had circulatory disorders in the vasa vasorum vascular system. Hypertrophy and sclerosis of the arteries of the vasa vasorum system, the narrowing of their lumen, and, as a result, impaired blood supply to the aortic wall were found in 34 patients with hyper-tension. In 18 patients (both in patients with hypertension and in patients without hypertension), T cells that adhered to the vasa vasorum endothelium were found. For the adhesion of blood mononuclear cells to the endothelium, the dysfunction of this endothelium is necessary, which occurs, in particular, when blood flow through the vessel is disturbed. It can be assumed that these 18 patients also have impaired blood circulation through the vasa vasorum system. In nine cases, massive infiltrates of T cells and macrophages were found, which surrounded and squeezed the vasa vasorum, preventing blood circulation. In six patients, parietal and obturating blood clots were found in the vasa vasorum vessels, which disrupted the normal blood supply to the aortic wall.

Thus, this pathology, or another, in the vessels of the vasa vasorum system was detected in all patients with thoracic aortic aneurysm. We believe that this indicates the importance of the state of the vessels of the vasa vasorum in the development of aortic aneurysm. In addition, pathological changes in these vessels may not always play a primary role, but always a very important role in the pathogenesis of this disease.

## Figures and Tables

**Table 1:** Comparative characteristics of adhesive T cells to the vasa vasorum.

Group Number	* Number of Patients	Number of Adhesive T Cells on the Vasa Vasorum	Comparison of T Cells in Groups 1 and 2	Comparison of T Cells in Groups 1 and 3	Comparison of T Cells in Groups 2 and 3
1.	9	44			
2.	14	16			
3.	25	5	*p* < 0.0794	*p* < 0.0006	*p* < 0.124

* One patient corresponded to one biopsy.

## Data Availability

Not applicable.
